# Association Between Neighborhood Disadvantage and Fertility Among Pregnancy Planners in the US

**DOI:** 10.1001/jamanetworkopen.2022.18738

**Published:** 2022-06-30

**Authors:** Mary D. Willis, Olivia R. Orta, Collette Ncube, Amelia K. Wesselink, Lan N. Đoàn, Kipruto Kirwa, Renée Boynton-Jarrett, Elizabeth E. Hatch, Lauren A. Wise

**Affiliations:** 1 School of Biological and Population Health Sciences, College of Public Health and Human Sciences, Oregon State University, Corvallis; 2 Department of Epidemiology, School of Public Health, Boston University, Boston, Massachusetts; 3 John Jay College of Criminal Justice, City University of New York, New York, New York; 4 Department of Population Health, Section for Health Equity, Grossman School of Medicine, New York University, New York; 5 Department of Public Health and Community Medicine, School of Medicine, Tufts University, Boston, Massachusetts; 6 Department of Pediatrics, School of Medicine, Boston University, Boston, Massachusetts

## Abstract

**Importance:**

Decades of inequitable policies in the US have yielded disparities in neighborhood quality, and some studies show that living in a socioeconomically disadvantaged neighborhood is associated with worse health outcomes, including reproductive health outcomes. However, no US studies to date have directly examined the association between residence in disadvantaged neighborhoods and fertility.

**Objective:**

To examine the association between residence in disadvantaged neighborhoods and fecundability, a sensitive marker of fertility with many health implications.

**Design, Setting, and Participants:**

This prospective preconception cohort study used the Pregnancy Study Online, for which baseline data were collected from June 19, 2013, through April 12, 2019. The study included 6356 participants who identified as female, were 21 to 45 years of age, were attempting conception without fertility treatment, and provided a valid residential address in the contiguous US at enrollment.

**Exposures:**

A standardized area deprivation index (ADI) derived at the census block group level applied to each residential address.

**Main Outcomes and Measures:**

Fecundability, the per-cycle probability of conception, via questionnaires that were completed every 8 weeks for 12 months, until conception or a censoring event. Proportional probabilities models were used to estimate fecundability ratios and 95% CIs for associations between ADI and fecundability. Restricted cubic splines were also implemented to examine nonlinearity. Models were adjusted for demographic characteristics and factors associated with fertility. The study’s a priori hypothesis was that higher levels of neighborhood disadvantage would be associated with decreased fecundability.

**Results:**

Among 6356 participants, 3725 pregnancies were observed for 27 427 menstrual cycles of follow-up. The mean (SD) baseline age was 30.0 (4.1) years, and most participants were non-Hispanic White (5297 [83.3%]) and nulliparous (4179 [65.7%]). Comparing the top and bottom deciles of disadvantaged neighborhood status, adjusted fecundability ratios were 0.79 (95% CI, 0.66-0.96) for national-level ADI rankings and 0.77 (95% CI, 0.65-0.92) for within-state ADI rankings. Restricted cubic splines showed some evidence of nonlinearity in the association. Associations were slightly stronger among participants with lower annual incomes (<$50 000).

**Conclusions and Relevance:**

In this cohort study, residence in a socioeconomically disadvantaged neighborhood was associated with moderately decreased fecundability. If confirmed in other studies, these results suggest that investments to reduce disadvantaged neighborhood status may yield positive cobenefits for fertility.

## Key Points

**Question:**

Is living in a disadvantaged neighborhood associated with decreased fertility?

**Findings:**

In this cohort study of 6356 women attempting to conceive without the use of fertility treatment, the probability of conception was reduced 21% to 23% per menstrual cycle when comparing the highest with the lowest deciles of disadvantaged neighborhoods. When disadvantaged neighborhood status was categorized within each state (as opposed to nationally), the results were slightly larger in magnitude.

**Meaning:**

These findings suggest that investments in disadvantaged neighborhoods may yield positive cobenefits for fertility.

## Teaser

This cohort study examines the association between disadvantaged neighborhood status and fecundability at the state and national levels among women attempting to conceive a child without the use of fertility treatment.

## Introduction

In the US, 10% to 15% of reproductive-aged couples experience infertility, defined as the inability to conceive after 12 months of unprotected intercourse.^1^ The economic burden of infertility is substantial, with health care costs that exceed $5 billion annually.^2^ Likewise, infertility is associated with significant psychosocial consequences.^2,3^ To date, there are few established modifiable risk factors to improve fecundability (a couple-based metric of the probability of conception during a menstrual cycle).

Infertility prevention is often discussed with respect to personal behavioral changes that may improve the probability of conception. However, structural, political, and environmental factors may also play a substantial role in fertility. A confluence of these factors is present in a socioeconomically disadvantaged neighborhood environment, defined by the mixture of educational attainment, employment status, household income, and housing quality in a community.^4,5^ A growing body of literature demonstrates associations between a disadvantaged neighborhood environment and adverse reproductive health outcomes.^6,7,8,9^ One standardized approach for measuring a relative disadvantaged neighborhood environment is the area deprivation index (ADI).^4,10^ Greater neighborhood disadvantage, as measured by the ADI, have been consistently associated with adverse health outcomes, such as hospital readmissions.^11^ We hypothesized a similar association between a disadvantaged neighborhood environment and fecundability.

**Figure 1.**
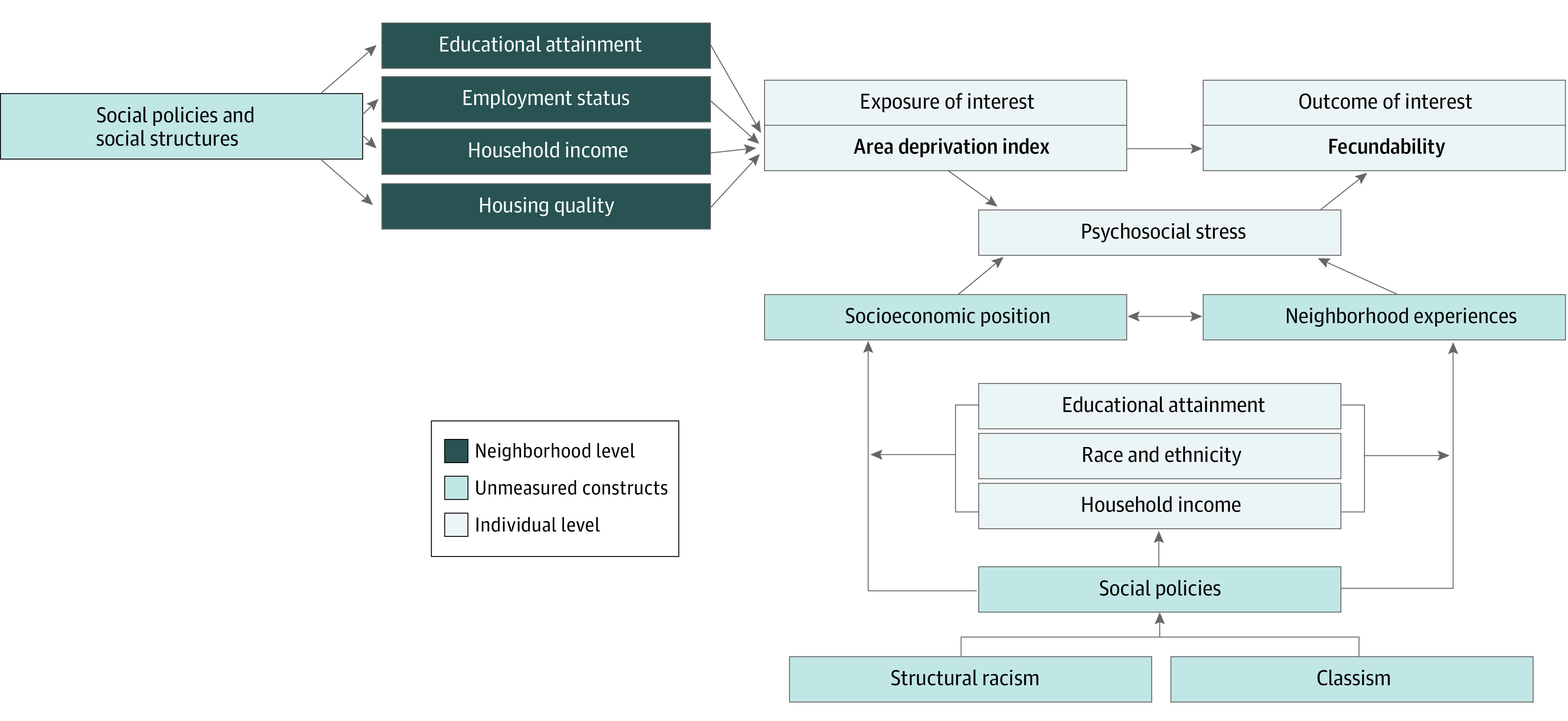
Conceptual Diagram of the Hypothesized Associations Between Resource-Poor Neighborhood Environment and Fecundability With Respect to Individual and Neighborhood Factors

There are multiple pathways by which neighborhood disadvantage may reduce fecundability (Figure 1). Residing in a socioeconomically disadvantaged neighborhood is associated with increased perceived stress,^12^ cortisol levels,^13^ and allostatic load.^14^ Neighborhood exposures are generally chronic, because most people infrequently relocate.^15^ Moreover, members of racial and ethnic minority groups are less likely to move to high-opportunity neighborhoods (ie, communities with high opportunity for upward socioeconomic mobility).^16,17^ Perceived stress has been associated with poorer in vitro fertilization outcomes^18^ and reduced fecundability among couples attempting spontaneous conception.^19,20^ Similar results have also been found for salivary α-amylase, a biomarker of stress levels, and fecundability.^21,22^ To our knowledge, no studies of disadvantaged neighborhood environment have directly examined fertility-related outcomes in the US.

State economic policies may particularly influence the day-to-day lives of its residents, including programs that can exacerbate or mitigate socioeconomic disparities.^23^ Many policies have measured effects on health, such as improved life expectancy in states with health-protective programs (eg, policies that support civil rights, promote the environment, and invest in underresourced communities.)^24^ However, members of marginalized racial and ethnic minority groups are more likely to reside in socioeconomically disadvantaged neighborhoods owing to structural racism enacted through residential segregation, systematic disinvestment in neighborhood infrastructure, and associated disparate exposure to environmental toxicants.^25,26,27,28^ Equitably implemented policies to improve disadvantaged neighborhoods may have positive benefits for health outcomes that have strong sociodemographic disparities, including fertility.^29,30,31^

In this US prospective cohort study of couples attempting pregnancy, we examined the association between residence in a disadvantaged neighborhood and fecundability. We classified disadvantaged neighborhood status at the national level (ie, ranking within the nation) and state level (ie, ranking within its own state) to understand contextual variation.

## Methods

### Study Population

Pregnancy Study Online (PRESTO) is an online preconception cohort study of couples attempting spontaneous conception.^32^ Briefly, eligible participants identified as female and were 21 to 45 years of age, residents of the US or Canada, and attempting conception without fertility treatment. Participants provided written informed consent and completed a baseline questionnaire of sociodemographic and lifestyle characteristics, medical history, medication use, and reproductive health. Every 8 weeks for up to 12 months, participants answered follow-up questionnaires on menstrual cycle characteristics and pregnancy status. The study protocol was approved by the institutional review board at the Boston University Medical Campus, Boston, Massachusetts. We followed the Strengthening the Reporting of Observational Studies in Epidemiology (STROBE) reporting guideline.

From June 19, 2013, through April 12, 2019, 10 293 eligible female participants completed the baseline questionnaire. We excluded 200 participants with implausible data related to their baseline last menstrual period (LMP), 2055 who had been attempting to conceive for 6 or more cycles at study entry, 1310 who lived in Canada, 56 who resided outside the contiguous US, 211 with a baseline address that could not be accurately geocoded, and 105 whose census block group lacked an ADI score. These exclusions yielded an analytic sample of 6356 participants.

### Area Deprivation Exposure Assessment

We used the 2015 ADI to estimate limitations of neighborhood disadvantage.^4,10,11^ Briefly, the ADI was constructed using sociodemographic data from the 2011-2015 American Community Survey 5-year means, and the score includes population indicators related to educational attainment, housing, employment, and poverty.^4,10^ Higher scores represent neighborhoods with greater disadvantage. We obtained 2 ADI scores for each census block group: national percentile ranking (1-100) and within-state decile ranking (1-10). We hypothesized that the within-state ADI would be more strongly associated with fecundability because it reduces confounding from dissimilar state contexts (eg, social policy, cost of living). Using participants’ geocoded baseline addresses, we assigned each residential address to its corresponding census block group and linked it to its ADI score.

### Fecundability Outcome Assessment

On the baseline questionnaire, participants reported the number of menstrual cycles during which they had been attempting conception (“For how many cycles have you been trying to get pregnant?”) and whether they had regular menstrual cycles (“Within the past couple of years, has your menstrual period been regular?”). On the baseline and follow-up questionnaires, participants provided their LMP date. Participants who reported that their cycles were regular (ie, predictable within a few days) were asked about their typical cycle length. Participants with irregular cycles were asked for the number of menses per year and the estimated number of days until their next menses, which we used to estimate cycle length.

Every 8 weeks after enrollment, participants completed follow-up questionnaires on which they reported current pregnancy status, pregnancy losses, cessation of pregnancy attempt, or initiation of fertility treatment. If participants reported a conception, we asked how their pregnancy was confirmed (ie, home pregnancy test, gynecologic examination, blood test). For nonpregnant participants, we inquired whether they were still trying to conceive. Among those lost to follow-up, we ascertained outcome information in several ways: (1) contacting participants directly via telephone or email, (2) searching the internet for baby registries and birth announcements, and (3) linking with birth registries in selected states (California, Florida, Massachusetts, Michigan, New York, Ohio, Pennsylvania, and Texas). Participants contributed cycles at risk until pregnancy, regardless of its outcome, or a censoring event, including cessation of pregnancy attempt (164 [2.6%]), initiation of fertility treatment (437 [6.9%]), loss to follow-up or withdrawal (1019 [16.0%]), or 12 cycles (1137 [17.9%]), whichever occurred first. We calculated time to pregnancy in discrete menstrual cycles using the following formula:

Cycles Trying to Conceive at Study Entry + [(LMP Date From Most Recent Follow-up Questionnaire − Date of Baseline Questionnaire) / Cycle Length] + 1

### Covariate Assessment

On the baseline questionnaire, we ascertained sociodemographic data (age, educational attainment, annual household income, and race and ethnicity), reproductive and contraceptive history (parity, history of infertility, last method of contraception), daily multivitamin/folic acid use, and factors related to intensity of trying to conceive (intercourse frequency, doing something to improve chances of conception [eg, charting menstrual cycles]). Racial and ethnic data were collected to account for the historical marginalization of some populations, including disparities in fertility and subsequent access to fertility treatment. For national and state-level ADI rankings, individual sociodemographic characteristics are standardized to age distribution of the cohort at baseline, with the exception of age.

### Statistical Analysis

#### Main Analysis

Data were analyzed from October 1, 2019, to May 24, 2022. We used the Andersen-Gill data structure to account for left truncation owing to delayed entry into the risk set.^33^ We used proportional probabilities regression models to calculate fecundability ratios (FRs) and 95% CIs comparing each category of exposure with the reference group (ie, polytomous categorical exposure by decile using indicator variables). All models included indicator variables for menstrual cycle at risk to account for the decline in population fecundability with increasing attempt time. We also fit restricted cubic splines to examine the potential for nonlinear associations between ADI and fecundability.

In adjusted models, we controlled for clinical factors associated with fertility as precision variables, including participant age (<25, 25-29, 30-34, 35-39, or ≥40 years), daily multivitamin or folic acid intake, parity (nulliparous or parous), intercourse frequency (<1, 1, 2-3, or ≥4 times/week), last method of contraception used (oral contraceptives, other hormonal contraceptives, barrier methods, withdrawal, rhythm, and/or other methods), doing something to improve chances of conception (ie, timing intercourse and/or ovulation testing), and year of enrollment (2013 through 2019). We hypothesized that individual demographic and socioeconomic attributes (eg, educational attainment, income, race and ethnicity) may act along the causal pathway of the association between ADI and fecundability more strongly than operating as confounders; therefore, we did not adjust for these characteristics in the primary adjusted model, although we explored their influence in sensitivity analyses, described hereinafter.

We used multiple imputation to impute missing data on exposure, outcome, and covariates with fully conditional specifications methods and statistically combined 20 data sets to generate FRs and 95% CIs.^34^ Women without follow-up questionnaires (758 [11.9%]) were assigned 1 cycle of follow-up, with pregnancy status multiply imputed at that cycle. Missingness ranged from 0 (eg, age) to 3% (household income). All statistical analyses were performed using SAS version 9.4 (SAS Institute Inc).

#### Sociodemographic Subgroup Analysis

We stratified by individual demographic and socioeconomic variables that could modify the association between disadvantaged neighborhood environment at the state level and fecundability, including annual household income (<$50 000/y vs ≥$150 000/y) and educational attainment (<16 vs ≥16 years, corresponding to college graduates). The sample size did not allow for reasonable disaggregation by racial and ethnic identity.

#### Sensitivity Analysis

We conducted several sensitivity analyses. First, we restricted the analysis to participants who reported fewer than 3 cycles of attempt time at enrollment. Participants with longer attempt times at enrollment may have changed their behaviors to increase chances of conception^35^ and may report their attempt time with more error.^32^ This restriction also minimizes selection bias if participation in this study was related to a disadvantaged neighborhood environment and subfertility. Second, we stratified results by parity (nulliparous or parous), because parous participants with adequate financial resources may be more likely to change residences after having children. Third, we evaluated the influence of controlling for selected individual socioeconomic indicators in the adjusted regression model: race and ethnicity, educational attainment, and household income.

## Results

### Descriptive Statistics

Among the 6356 participants included in the analysis, we observed 3725 pregnancies during 27 427 menstrual cycles of follow-up, spanning 6024 block groups across the 48 contiguous US states. Mean (SD) baseline age was 30.0 (4.1) years. Most participants were non-Hispanic White (5297 [83.3%]), nulliparous (4179 [65.7%]), and had at least 16 years of education (4611 [72.5%]). Participants had greater mean annual household income than the general population, although the range included lower-income (<$50 000/y; 1341 [21.1%]) and higher-income (≥$150 000/y; 1030 [16.2%]) groups (Table 1).

**Table 1.** Baseline Characteristics of 6356 Pregnancy Study Online Participants by Area Deprivation Index Rankings

Characteristic	Participant group^a^						
	All	National ADI ranking			Within-state ADI ranking		
		Low (<33)	Middle (33-66)	High (≥67)	Low (<3)	Middle (3-7)	High (≥8)
All	6356 (100)	2722 (42.8)	2419 (38.0)	1115 (17.5)	1676 (26.4)	3428 (53.9)	1252 (19.7)
Age, mean (SD), y	30.0 (4.1)	30.8 (3.9)	29.6 (4.1)	28.9 (4.4)	30.7 (3.9)	29.9 (4.1)	29.1 (4.3)
Annual household income							
<$50 000	1341 (21.1)	241 (10.8)	564 (22.6)	536 (41.6)	148 (10.6)	711 (20.7)	482 (36.1)
≥$150 000	1030 (16.2)	778 (26.3)	213 (9.2)	39 (3.6)	515 (28.2)	452 (13.3)	63 (5.5)
Educational attainment							
High school or less	342 (5.4)	47 (2.0)	141 (5.7)	154 (12.0)	31 (2.2)	167 (4.9)	144 (10.8)
Bachelor’s degree or more	4611 (72.5)	2328 (83.7)	1680 (69.9)	603 (51.4)	1443 (84.4)	2486 (72.6)	682 (56.1)
Race and ethnicity							
Hispanic/Latina	463 (7.3)	196 (7.5)	167 (6.8)	100 (8.0)	94 (5.8)	244 (7.1)	125 (9.8)
Non-Hispanic Asian	114 (1.8)	78 (2.6)	22 (0.9)	14 (1.4)	44 (2.4)	49 (1.5)	21 (1.9)
Non-Hispanic Black	222 (3.5)	46 (1.7)	77 (3.3)	99 (8.9)	26 (1.5)	107 (3.2)	89 (7.5)
Non-Hispanic White	5297 (83.3)	2313 (84.7)	2046 (84.4)	938 (76.5)	1449 (86.7)	2895 (84.1)	953 (75.6)
Multiple races or other race^b^	260 (4.1)	89 (3.5)	107 (4.5)	64 (5.3)	63 (3.9)	133 (3.9)	64 (5.3)
Current smoker	666 (10.5)	147 (5.7)	288 (11.8)	231 (18.3)	89 (5.8)	383 (11.2)	194 (14.8)
Last contraception method used was oral contraceptive pills	2157 (33.9)	948 (35.5)	826 (34.1)	383 (30.8)	576 (34.9)	1170 (34.1)	411 (32.3)
Intercourse <1 time/week	1371 (21.6)	622 (21.8)	510 (21.5)	239 (20.7)	380 (21.4)	741 (21.7)	250 (20.9)
Doing something to improve chances of conception	4912 (77.3)	2126 (77.8)	1866 (77.3)	920 (75.6)	1333 (79.5)	2627 (76.7)	952 (76.2)
Nulliparous	4179 (65.7)	1906 (71.6)	1581 (64.7)	692 (55.3)	1154 (70.3)	2258 (65.6)	767 (60.2)
Multivitamin use	5090 (80.1)	2322 (84.4)	1903 (79.0)	865 (72.1)	1426 (84.3)	2757 (80.4)	907 (73.3)
US region							
Northeast	1755 (27.6)	1124 (40.5)	463 (19.2)	168 (14.3)	412 (23.8)	969 (28.4)	374 (30.8)
Midwest	1596 (25.1)	396 (14.7)	773 (31.8)	427 (35.1)	481 (29.1)	823 (23.9)	292 (23.2)
South	1828 (28.8)	564 (20.9)	807 (33.5)	457 (37.7)	525 (31.9)	1015 (29.6)	288 (22.7)
West	1177 (18.5)	638 (23.9)	376 (15.5)	163 (12.9)	258 (15.3)	621 (18.2)	298 (23.3)

Abbreviation: ADI, area deprivation index.

^a^ Unless indicated otherwise, data are expressed as No. (%) of participants. All percentages are standardized to the age distribution of the cohort at baseline, with the exception of age.

^b^ Includes American Indian and Alaska Native, and non-Hispanic multiple or other races.

At the national level, participants residing in disadvantaged neighborhoods reported younger age (mean [SD], 28.9 [4.4] years), lower educational attainment (≤12 years, 603 [51.4%]), and lower household income (<50 000, 536 [41.6%]); were more likely to smoke (231 [18.3%]); and were less likely to identify as non-Hispanic White (938 [76.5%]) (Table 1). Characteristics were similar at the within-state level, although the magnitude of differences was attenuated. The Spearman correlation between the national and within-state ADI rankings was 0.76.

### Main Statistical Analysis

For the nationally ranked ADI, we observed an inverse association between the ADI and fecundability among participants who resided in a neighborhood with an ADI greater than 60. For instance, the FR comparing the most disadvantaged neighborhoods (national ADI, 91-100) with the least disadvantaged neighborhoods (national ADI, 1-10) was 0.81 (95% CI, 0.67-0.98) in unadjusted models and 0.79 (95% CI, 0.66-0.96) in adjusted models (Table 2). For the restricted cubic splines for the national-level ADI metric, we observed an approximately linear inverse association. These results correspond to a 19% and 21% reduction in fecundability, respectively.

**Table 2.** Associations Between Area Deprivation Index and Fecundability, Pregnancy Study Online

Exposure	No. of pregnancies	No. of cycles	FR (95% CI)	
			Model 1^a^	Model 2^b^
**National ADI categorical ranking**				
≤10 (least disadvantaged)	468	3175	1 [Reference]	1 [Reference]
11-20	629	4223	1.03 (0.92-1.15)	0.99 (0.89-1.10)
21-30	543	3865	0.98 (0.87-1.10)	0.93 (0.83-1.04)
31-40	517	3745	0.96 (0.86-1.08)	0.92 (0.82-1.04)
41-50	437	3249	0.94 (0.83-1.06)	0.89 (0.78-1.00)
51-60	370	2618	0.98 (0.86-1.11)	0.92 (0.83-1.04)
61-70	276	2243	0.89 (0.77-1.02)	0.82 (0.72-0.95)
71-80	189	1821	0.75 (0.64-0.88)	0.70 (0.60-0.82)
81-90	163	1338	0.86 (0.73-1.02)	0.83 (0.70-0.98)
>90 (most disadvantaged)	133	1150	0.81 (0.67-0.98)	0.79 (0.66-0.96)
**Within-state ADI categorical ranking**				
1 (least disadvantaged)	483	3163	1 [Reference]	1 [Reference]
2	584	4068	0.99 (0.88-1.10)	0.98 (0.88-1.10)
3	546	3789	0.98 (0.87-1.10)	0.97 (0.87-1.09)
4	453	3158	0.96 (0.86-1.09)	0.95 (0.84-1.07)
5	403	2909	0.95 (0.84-1.08)	0.95 (0.84-1.07)
6	311	2596	0.84 (0.74-0.97)	0.84 (0.73-0.96)
7	288	2374	0.85 (0.74-0.98)	0.85 (0.74-0.98)
8	284	2099	0.95 (0.82-1.08)	0.94 (0.82-1.08)
9	210	1746	0.85 (0.73-0.98)	0.84 (0.72-0.98)
10 (most disadvantaged)	163	1537	0.75 (0.63-0.90)	0.77 (0.65-0.92)

Abbreviations: ADI, area deprivation index; FR, fecundability ratio.

^a^ Unadjusted.

^b^ Adjusted for age, daily multivitamin or folic acid intake, parity, intercourse frequency, last method of contraception used before attempting pregnancy, doing something to improve chances of conception, and year of baseline enrollment.

**Figure 2.**
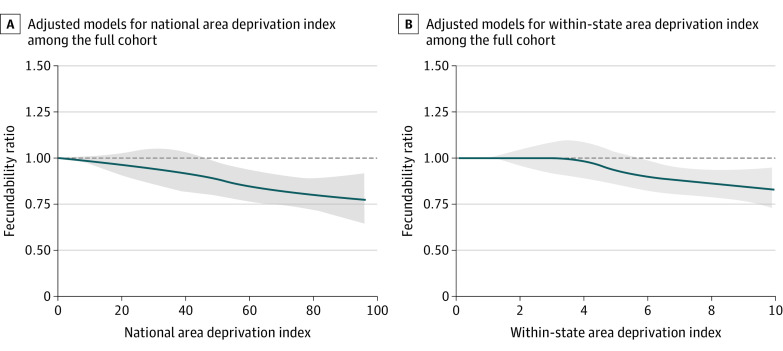
Restricted Cubic Splines Between the Area Deprivation Index and Fecundability Graphs are plots of restricted cubic splines, where the observations are trimmed at the first and 99th percentiles. The reference value is the minimum. The knots are located at 20, 40, 60, and 80 in the national spline and 2, 4, 6, and 8 in within-state spline. The blue line indicates fecundability ratio; the shaded gray area, 95% CI. Adjusted regressions contain covariates for age, daily multivitamin or folic acid intake, parity, intercourse frequency, last method of contraception used before attempting pregnancy, doing something to improve the chances of conception, and year of baseline enrollment.

At the state-level ranking, we found an inverse association between the ADI and fecundability among the participants who resided in a neighborhood with an ADI greater than 5. When comparing the most disadvanted neighborhoods (state-level ADI, 10) with the least disadvantaged neighborhoods (state-level ADI, 1), the FR was 0.75 (95% CI, 0.63-0.90) in unadjusted models and 0.77 (95% CI, 0.65-0.92) in adjusted models, corresponding to a 25% and 23% reduction inf fecundability, respectively (Table 2). For the restricted cubic splines for the state-level ADI metric, we observed little evidence of an association below 4 and an approximately linear association beyond 4 (Figure 2).

### Sociodemographic Subgroup Analyses

We performed several subgroup analyses where we exclusively focused on the state-level ADI ranking (eFigures 1 and 2 in the Supplement). When we stratified by educational attainment, the spline curve was highly attenuated for college graduates compared with the primary model results, whereas there was a largely flat curve with less precision among participants who did not graduate from college. Among participants with annual household incomes less than $50 000, the spline curve showed an inverse association between ADI and fecundability, although imprecise. In contrast, among those with annual household incomes of $150 000 or more, the spline curve was mostly flat.

### Sensitivity Analysis

Among participants with fewer than 3 cycles of pregnancy attempt at enrollment, we observed similar results with less precision (eTable 1 in the Supplement). Results were similar across strata of parity, but inverse associations were less precise among parous participants (eTable 2 in the Supplement). When we evaluated the addition of individual socioeconomic variables, adjusting for race and ethnicity and educational attainment yielded partially attenuated results, although the 95% CIs were wider (eTable 3 in the Supplement). Adding household income strongly attenuated the overall association.

## Discussion

Using data from a US preconception cohort study, greater limitations of neighborhood disadvantage were associated with reduced fecundability, a couple-based metric of the per-cycle probability of conception. Associations between ADI and fecundability were similar when neighborhood disadvantage was measured relative to the nation or the state, which lends support for the hypothesis that local context may be particularly influential in fertility.

This finding is consistent with literature demonstrating that neighborhood socioeconomic environments influence reproductive health.^6,7,8,9^ Studies using vital statistics records (ie, birth certificates) show that a disadvantaged neighborhood environment is associated with comorbidities during pregnancy, such as increased risks of gestational hypertension (risk ratio for lowest vs highest quartile: 1.24 [95% CI, 1.14-1.35])^6^ and inadequate gestational weight gain (relative risk for lowest vs highest quartile: 1.1 [95% CI, 1.1-1.2]).^8^ Similar increased risks of preterm birth were found among non-Hispanic Black and White women by neighborhood disadvantage levels,^9^ but there is substantial geographic variation across the US in the magnitude of the association.^7,9^ Other work in North America has shown that lower neighborhood household income is a large driver of the association between a disadvantaged neighborhood environment and lower birth weight^36,37^ and potentially stillbirth.^37^ However, these studies used slightly different metrics of disadvantaged neighborhood environment, precluding a direct comparison of results.

Neighborhood context, including a socioeconomicly disadvantaged neighborhood environment, is a complex phenomenon that encompasses a variety of chronic stress exposure pathways. Public policies influencing decisions about neighborhood investment and disinvestment (eg, redlining) may perpetuate a cycle of chronic stress and continued neighborhood socioeconomic disadvantage.^25,38^ For example, higher neighborhood unemployment has been associated with decreased population-level fertility rates.^39^ Less affluent areas often have higher concentrations of air pollution,^40^ which has been associated with adverse fertility outcomes.^41,42,43,44,45,46,47^ There also may be fewer amenities such as green spaces to provide capacity restoration for residents, such as reducing stress.^26,48^ Conversely, urban renewal programs show positive health benefits for local populations,^29^ although the effects are mixed when gentrification is taken into account.^49^ Our results lend credibility to this hypothesis, because a disadvantaged neighborhood environment was associated with a modest decrease in fecundability in the most disadvantaged areas.

A key challenge in analyzing social determinants of health is deciding when individual sociodemographic factors (eg, race and ethnicity, income, educational attainment) should be incorporated into statistical models. Previous work in PRESTO showed that individuals’ socioeconomic factors ascertained in adulthood, such as lower educational attainment and household income, are associated with decreased fecundability.^50^ Both income and educational attainment may be influenced by neighborhood disadvantage earlier in life^51^; therefore, they are potential mediators of the exposure-outcome association. However, the present study examines neighborhood disadvantage in adulthood. To the extent that we assume people are socially immobile, we could assume similar ADI levels over time, so we could argue that educational attainment and income in adulthood are mediators. If, however, we assume a noteworthy change in neighborhood context with the transition from adolescence to adulthood, it is more likely that these socioeconomic attributes, along with race and ethnicity, influence the neighborhoods in which people live as adults, although these residential locations may not be by choice.^52,53,54,55^ Thus, the appropriateness of adjusting for individual socioeconomic factors depends largely on the conceptual framework, and disentangling the influence of individual vs neighborhood factors may not be possible. Careful consideration must be given toward the inclusion of these variables from health-related models when estimating the effects of a disadvantaged neighborhood environment, or similar spatial epidemiologic measures. Our conceptual framework, in combination with our sensitivity models, suggest that these socioeconomic attributes may be operating as mediators in this context.

### Limitations

This study has some limitations. We assessed exposures based on participants’ residential address and matched them to a corresponding census block group. Although the US Census aims for block groups to accurately represent neighborhoods, we do not know how participants interact with their surroundings, including time spent in nearby neighborhoods that may be ranked differently.^56,57,58^ We also cannot consider time-activity patterns, housing characteristics, local pollution, or other factors that may yield differences between our exposure metric and participants’ true neighborhood exposures. However, the prospective study design allows us to capture residential addresses before pregnancy is observed; thus, exposure misclassification is likely nondifferential with respect to the outcome.

We calculated fecundability using a combination of self-reported variables: pregnancy attempt time at study entry, LMP dates, usual cycle length, and pregnancy status. Each variable is measured with error, yielding some degree of potential outcome misclassification. However, previous work in PRESTO^32,59^ has validated LMP dates and usual cycle length and shown that mean gestational age of pregnancy detection is 4 weeks (ie, before a missed period). Therefore, we have evidence that participants are accurately reporting outcome data. Analyses restricted to participants with fewer than 3 cycles of attempted pregnancy at enrollment yield results similar to those of the main model. Thus, we do not anticipate that outcome misclassification is a large source of bias.

The composition of PRESTO’s study population, pregnancy planners who enroll in a prospective cohort via the internet, is unique. Based on a prior validation study,^60^ internet-based recruitment methods did not indicate biased etiologic associations. However, pregnancy planners may fundamentally differ from the general population at risk of pregnancy. Many PRESTO participants also have higher socioeconomic status compared with the general US population, per their household income and educational attainment.^50,61^ Therefore, our results may not be generalizable to populations of lower socioeconomic status. Future research should include racially and ethnically diverse populations across the socioeconomic spectrum to better understand fecundability disparities.

## Conclusions

Our findings underscore the importance of understanding the association between a disadvantaged neighborhood environment and reproductive health. In this study, residence in the most disadvantaged neighborhoods of the US was associated with reduced fecundability, a sensitive metric of fertility. If confirmed in other studies, our results suggest that policies and programs that address socioeconomic inequities may reduce infertility in local communities.

## Notes

Supplement. **eFigure 1.** Restricted Cubic Splines for Association Between Area Deprivation Index and Fecundability by Educational Attainment, Pregnancy Study Online (PRESTO) Participants **eFigure 2.** Restricted Cubic Splines for Association Between Area Deprivation Index and Fecundability by Household Income, Pregnancy Study Online (PRESTO) Participants **eTable 1.** Associations Between the Area Deprivation Index and Fecundability, Pregnancy Study Online (PRESTO) Participants With Fewer Than 3 Cycles of Pregnancy Attempt Time at Enrollment **eTable 2.** Associations Between the Area Deprivation Index and Fecundability by Parity, Pregnancy Study Online (PRESTO) Participants **eTable 3.** Alternative Model Iterations for Associations Between Area Deprivation Index and Fecundability, Pregnancy Study Online (PRESTO)
