# Supplementary material for: Association Between Neighborhood Disadvantage and Fertility Among Pregnancy Planners in the US
Source: JAMA Netw Open. 2022 Jun 30;5(6):e2218738. doi: 10.1001/jamanetworkopen.2022.18738 (PMC9247730; doi:10.1001/jamanetworkopen.2022.18738)
Supplement: Supplement. — eFigure 1. Restricted Cubic Splines for Association Between Area Deprivation Index and Fecundability by Educational Attainment, Pregnancy Study Online (PRESTO) Participants eFigure 2. Restricted Cubic Splines for Association Between Area Deprivation Index and Fecundability by Household Income, Pregnancy Study Online (PRESTO) Participants eTable 1. Associations Between the Area Deprivation Index and Fecundability, Pregnancy Study Online (PRESTO) Participants With Fewer Than 3 Cycles of Pregnancy Attempt Time at Enrollment eTable 2. Associations Between the Area Deprivation Index and Fecundability by Parity, Pregnancy Study Online (PRESTO) Participants eTable 3. Alternative Model Iterations for Associations Between Area Deprivation Index and Fecundability, Pregnancy Study Online (PRESTO) [file jamanetwopen-e2218738-s001.pdf]

Supplementary Online Content

Willis MD, Orta OR, Ncube C, et al. Association between neighborhood disadvantage and fertility among pregnancy planners in the US. *JAMA Netw Open*. 2022;5(6):e2218738. doi:10.1001/jamanetworkopen.2022.18738

eFigure 1. Restricted Cubic Splines for Association Between Area Deprivation Index and Fecundability by Educational Attainment, Pregnancy Study Online (PRESTO) Participants

eFigure 2. Restricted Cubic Splines for Association Between Area Deprivation Index and Fecundability by Household Income, Pregnancy Study Online (PRESTO) Participants

eTable 1. Associations Between the Area Deprivation Index and Fecundability, Pregnancy Study Online (PRESTO) Participants With Fewer Than 3 Cycles of Pregnancy Attempt Time at Enrollment

eTable 2. Associations Between the Area Deprivation Index and Fecundability by Parity, Pregnancy Study Online (PRESTO) Participants

eTable 3. Alternative Model Iterations for Associations Between Area Deprivation Index and Fecundability, Pregnancy Study Online (PRESTO)

This supplementary material has been provided by the authors to give readers additional information about their work.

© 2022 Willis MD et al. *JAMA Network Open*.

eFigure 1. Restricted Cubic Splines for Association Between Area Deprivation Index and Fecundability by Educational Attainment, Pregnancy Study Online (PRESTO) Participants

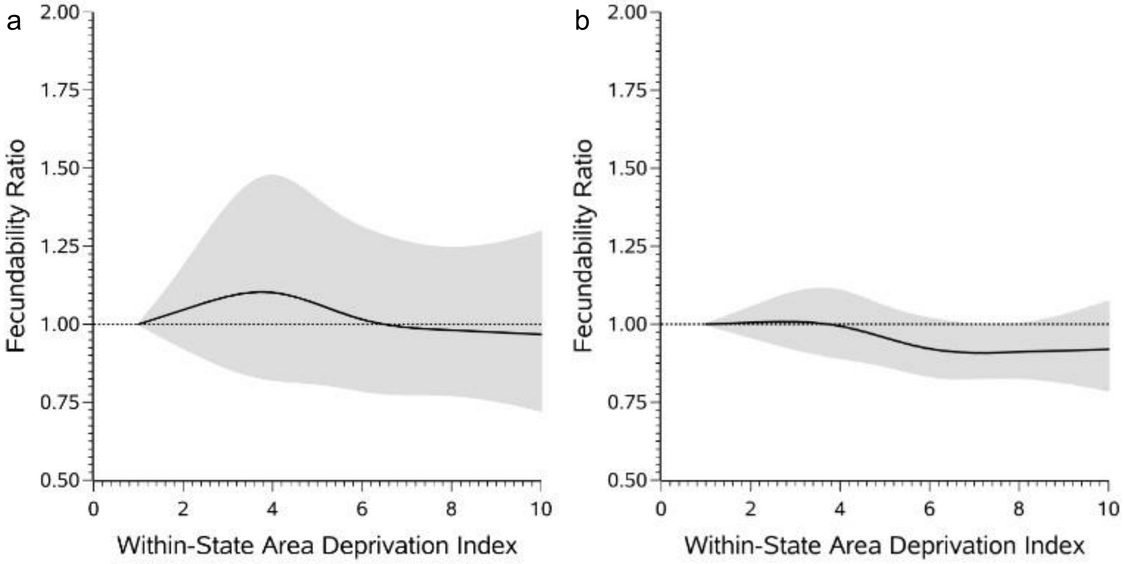

Graphs are plots of restricted cubic splines, where the observations are trimmed at the 1st and 99th percentiles. The reference value is 1 and the knots are located at 2, 4, 6, and 8 (black line=fecundability ratio, shaded grey area=95% confidence interval). Adjusted regressions contain covariates for age, daily multivitamin or folic acid intake, parity, intercourse frequency, last method of contraceptive use before attempting pregnancy, doing something to improve chances of conception (i.e., timing intercourse, charting menstrual cycles, ovulation predictor kit), and year of baseline enrollment. a) adjusted model restricted to participants who did not graduate from college (<16 years education); and b) adjusted model restricted to participants who are college graduates (≥16 years education).

© 2022 Willis MD et al. *JAMA Network Open*.

eFigure 2. Restricted Cubic Splines for Association Between Area Deprivation Index and Fecundability by Household Income, Pregnancy Study Online (PRESTO) Participants

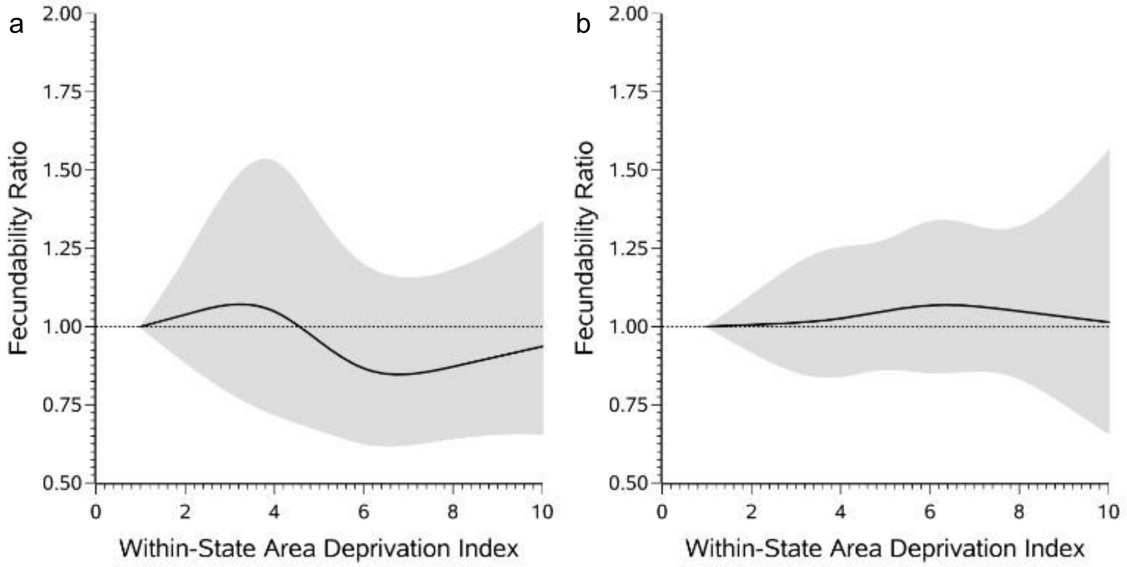

Graphs are plots of restricted cubic splines, where the observations are trimmed at the 1st and 99th percentiles. The reference value is 1 and the knots are located at 2, 4, 6, and 8 (black line=fecundability ratio, shaded grey area=95% confidence interval). Adjusted regressions contain covariates for age, daily multivitamin or folic acid intake, parity, intercourse frequency, last method of contraceptive use before attempting pregnancy, doing something to improve chances of conception (i.e., timing intercourse, charting menstrual cycles, ovulation predictor kit), and year of baseline enrollment. a) adjusted model for adjusted model restricted to participants with a household income <50,000 US dollars; and b) adjusted model restricted to participants with a household income ≥150,000 US dollars.

© 2022 Willis MD et al. *JAMA Network Open*.

eTable 1. Associations Between the Area Deprivation Index and Fecundability, Pregnancy Study Online (PRESTO) Participants With Fewer Than 3 Cycles of Pregnancy Attempt Time at Enrollment

Exposure	No. pregnancies	No. cycles	Model 1		Model 2	
			FR	95% CI	FR	95% CI
**National Area Deprivation Index Ranking**						
Categorical Ranking						
1-10 (Least deprived)	379	2,327	REF	REF	REF	REF
11-20	476	3,022	0.98	(0.87, 1.12)	0.97	(0.86, 1.10)
21-30	390	2,600	0.94	(0.82, 1.07)	0.92	(0.80, 1.04)
31-40	374	2,449	0.95	(0.83, 1.08)	0.97	(0.85, 1.10)
41-50	323	2,154	0.93	(0.81, 1.07)	0.92	(0.84, 1.06)
51-60	272	1,664	1.00	(0.86, 1.15)	0.97	(0.84, 1.12)
61-70	173	1,364	0.81	(0.68, 0.95)	0.79	(0.66, 0.93)
71-80	123	1,048	0.73	(0.60, 0.88)	0.75	(0.62, 0.91)
81-90	106	801	0.83	(0.68, 1.02)	0.85	(0.70, 1.05)
91-100 (Most deprived)	92	651	0.86	(0.69, 1.07)	0.88	(0.72, 1.10)
**Within-State Area Deprivation Index Ranking**						
Categorical Ranking						
1 (Least deprived)	384	2,359	REF	REF	REF	REF
2	439	2,754	1.01	(0.89, 1.14)	1.01	(0.89, 1.14)
3	408	2,544	1.01	(0.89, 1.15)	1.01	(0.89, 1.15)
4	324	2,180	0.94	(0.82, 1.08)	0.94	(0.82, 1.08)
5	277	1,883	0.94	(0.81, 1.08)	0.94	(0.82, 1.08)
6	218	1,595	0.86	(0.74, 1.01)	0.88	(0.75, 1.02)
7	205	1,465	0.89	(0.76, 1.04)	0.94	(0.80, 1.09)
8	196	1,293	0.97	(0.83, 1.13)	0.99	(0.84, 1.16)
9	153	1,083	0.90	(0.76, 1.07)	0.92	(0.77, 1.10)
10 (Most deprived)	104	924	0.72	(0.58, 0.89)	0.75	(0.60, 0.93)

Model 1 is unadjusted. Model 2 is adjusted for age, daily multivitamin or folic acid intake, parity, intercourse frequency, last method of contraceptive use before attempting pregnancy, doing something to improve chances of conception, and year of baseline enrollment.

© 2022 Willis MD et al. *JAMA Network Open*.

eTable 2. Associations Between the Area Deprivation Index and Fecundability by Parity, Pregnancy Study Online (PRESTO) Participants

Exposure	Nulliparous						Parous					
	No. pregnancies	No. cycles	Model 1		Model 2		No. pregnancies	No. cycles	Model 1		Model 2	
			FR	95% CI	FR	95% CI			FR	95% CI	FR	95% CI
**National Area Deprivation Index Ranking**												
Categorical Ranking												
1-10 (Least deprived)	344	2,423	REF	REF	REF	REF	124	752	REF	REF	REF	REF
11-20	414	3,056	0.98	(0.85, 1.11)	0.94	(0.82, 1.07)	215	1,167	1.12	(0.92, 1.36)	1.08	(0.89, 1.32)
21-30	377	2,763	0.98	(0.86, 1.13)	0.95	(0.83, 1.08)	166	1,102	0.95	(0.77, 1.17)	0.88	(0.71, 1.09)
31-40	332	2,572	0.94	(0.81, 1.08)	0.91	(0.79, 1.05)	185	1,173	0.98	(0.79, 1.20)	0.94	(0.76, 1.16)
41-50	277	2,251	0.90	(0.77, 1.04)	0.87	(0.75, 1.01)	160	998	0.99	(0.79, 1.23)	0.91	(0.73, 1.14)
51-60	249	1,769	1.01	(0.87, 1.18)	0.97	(0.83, 1.13)	121	849	0.88	(0.70, 1.11)	0.81	(0.64, 1.04)
61-70	167	1,488	0.84	(0.70, 1.00)	0.80	(0.67, 0.95)	109	755	0.93	(0.74, 1.18)	0.89	(0.69, 1.13)
71-80	100	1,016	0.73	(0.59, 0.90)	0.72	(0.58, 0.89)	89	805	0.73	(0.56, 0.94)	0.69	(0.53, 0.90)
81-90	92	772	0.86	(0.69, 1.07)	0.86	(0.69, 1.08)	71	566	0.82	(0.62, 1.07)	0.81	(0.61, 1.08)
91-100 (Most deprived)	74	735	0.76	(0.64, 0.97)	0.78	(0.61, 1.00)	59	415	0.86	(0.63, 1.16)	0.83	(0.61, 1.13)
**Within-State Area Deprivation Index Ranking**												
Categorical Ranking												
1-10 (Least deprived)	329	2,233	REF	REF	REF	REF	154	930	REF	REF	REF	REF
11-20	395	2,968	0.93	(0.81, 1.07)	0.92	(0.81, 1.06)	189	1,100	1.13	(0.93, 1.37)	1.12	(0.93, 1.37)
21-30	368	2,758	0.93	(0.81, 1.07)	0.91	(0.79, 1.05)	178	1,031	1.11	(0.91, 1.35)	1.12	(0.92, 1.37)
31-40	289	2,069	0.95	(0.82, 1.10)	0.93	(0.80, 1.08)	164	1,089	0.98	(0.80, 1.20)	0.97	(0.80, 1.19)
41-50	267	2,085	0.93	(0.80, 1.08)	0.92	(0.79, 1.07)	136	824	1.01	(0.82, 1.25)	1.03	(0.83, 1.27)
51-60	209	1,766	0.85	(0.72, 1.00)	0.84	(0.72, 0.99)	102	830	0.83	(0.66, 1.05)	0.85	(0.67, 1.07)
61-70	177	1,477	0.85	(0.72, 1.01)	0.88	(0.74, 1.04)	111	885	0.84	(0.67, 1.06)	0.84	(0.67, 1.06)
71-80	181	1,407	0.91	(0.77, 1.08)	0.92	(0.77, 1.09)	103	692	1.00	(0.79, 1.26)	1.02	(0.81, 1.29)
81-90	119	1,092	0.78	(0.64, 0.95)	0.77	(0.63, 0.94)	91	654	0.94	(0.74, 1.20)	0.98	(0.77, 1.26)
91-100 (Most deprived)	92	990	0.70	(0.56, 0.87)	0.73	(0.58, 0.91)	71	547	0.84	(0.64, 1.11)	0.91	(0.77, 1.26)

Model 1 is unadjusted. Model 2 is adjusted for age, daily multivitamin or folic acid intake, parity, intercourse frequency, last method of contraceptive use before attempting pregnancy, doing something to improve chances of conception, and year of baseline enrollment.

© 2022 Willis MD et al. *JAMA Network Open*.

eTable 3. Alternative Model Iterations for Associations Between Area Deprivation Index and Fecundability, Pregnancy Study Online (PRESTO)

Exposure	No. pregnancies	No. cycles	Model 1		Model 2		Model 3		Model 4		Model 5	
			FR	95% CI	FR	95% CI	FR	95% CI	FR	95% CI	FR	95% CI
**National Area Deprivation Index**												
Categorical Ranking												
1-10 (Least deprived)	468	3,175	REF	REF	REF	REF	REF	REF	REF	REF	REF	REF
11-20	629	4,223	1.03	(0.92, 1.15)	0.99	(0.89, 1.10)	0.98	(0.88, 1.09)	1.00	(0.90, 1.12)	1.00	(0.90, 1.12)
21-30	543	3,865	0.98	(0.87, 1.10)	0.93	(0.83, 1.04)	0.92	(0.82, 1.03)	0.94	(0.84, 1.06)	0.95	(0.85, 1.07)
31-40	543	3,745	0.96	(0.86, 1.08)	0.92	(0.82, 1.04)	0.91	(0.81, 1.03)	0.95	(0.85, 1.07)	0.96	(0.86, 1.09)
41-50	437	3,249	0.94	(0.83, 1.06)	0.89	(0.78, 1.00)	0.88	(0.78, 1.00)	0.94	(0.83, 1.06)	0.96	(0.84, 1.08)
51-60	370	2,618	0.98	(0.86, 1.11)	0.92	(0.83, 1.04)	0.91	(0.80, 1.03)	0.97	(0.86, 1.11)	1.00	(0.88, 1.15)
61-70	276	2,243	0.89	(0.77, 1.02)	0.82	(0.72, 0.95)	0.82	(0.71, 0.94)	0.89	(0.77, 1.03)	0.92	(0.80, 1.07)
71-80	189	1,821	0.75	(0.64, 0.88)	0.70	(0.60, 0.82)	0.70	(0.59, 0.82)	0.77	(0.66, 0.91)	0.80	(0.68, 0.95)
81-90	163	1,338	0.86	(0.73, 1.02)	0.83	(0.70, 0.98)	0.83	(0.70, 0.99)	0.94	(0.79, 1.12)	0.98	(0.82, 1.18)
91-100 (Most deprived)	133	1,150	0.81	(0.67, 0.98)	0.79	(0.66, 0.96)	0.82	(0.67, 0.99)	0.92	(0.76, 1.11)	0.96	(0.83, 1.16)
**Within-State Area Deprivation Index**												
Categorical Ranking												
1 (Least deprived)	483	3,163	REF	REF	REF	REF	REF	REF	REF	REF	REF	REF
2	584	4,068	0.99	(0.88, 1.10)	0.98	(0.88, 1.10)	0.98	(0.88, 1.10)	0.99	(0.89, 1.11)	1.00	(0.89, 1.12)
3	546	3,789	0.98	(0.87, 1.10)	0.97	(0.87, 1.09)	0.98	(0.87, 1.10)	1.01	(0.90, 1.13)	1.02	(0.91, 1.14)
4	453	3,158	0.96	(0.86, 1.09)	0.95	(0.84, 1.07)	0.95	(0.84, 1.07)	0.98	(0.87, 1.11)	1.00	(0.88, 1.13)
5	403	2,909	0.95	(0.84, 1.08)	0.95	(0.84, 1.07)	0.95	(0.84, 1.07)	0.99	(0.88, 1.12)	1.01	(0.89, 1.14)
6	311	2,596	0.84	(0.74, 0.97)	0.84	(0.73, 0.96)	0.84	(0.74, 0.96)	0.88	(0.77, 1.01)	0.90	(0.79, 1.03)
7	288	2,374	0.85	(0.74, 0.98)	0.85	(0.74, 0.98)	0.85	(0.74, 0.98)	0.91	(0.79, 1.04)	0.93	(0.81, 1.07)
8	284	2,099	0.95	(0.82, 1.08)	0.94	(0.82, 1.08)	0.95	(0.83, 1.08)	1.01	(0.88, 1.16)	1.05	(0.91, 1.20)
9	210	1,746	0.85	(0.73, 0.98)	0.84	(0.72, 0.98)	0.85	(0.73, 0.99)	0.95	(0.81, 1.10)	0.97	(0.83, 1.14)
10 (Most deprived)	163	1,537	0.75	(0.63, 0.90)	0.77	(0.65, 0.92)	0.79	(0.66, 0.94)	0.87	(0.73, 1.04)	0.91	(0.76, 1.08)

Model 1: unadjusted. Model 2: age, daily multivitamin or folic acid intake, parity, intercourse frequency, last method of contraceptive use before attempting pregnancy, doing something to improve chances of conception (i.e., timing intercourse, charting menstrual cycles, ovulation predictor kit), and year of baseline enrollment. Model 3: Added race/ethnicity. Model 4: Added race/ethnicity and educational attainment. Model 5: Added race/ethnicity, educational attainment, and household income.

© 2022 Willis MD et al. *JAMA Network Open*.
